# Supplementary material for: Dynamical networking of polymer networks with dedicated cross-linker particles
Source: Eur Phys J E Soft Matter. 2026 Apr 1;49(4):28. doi: 10.1140/epje/s10189-026-00568-w (PMC13043583; doi:10.1140/epje/s10189-026-00568-w)
Supplement: Supplementary file 1 — (pdf 4781 KB) [file 10189_2026_568_MOESM1_ESM.pdf]

Supplementary Information for: *Dynamical Networking of Polymer Networks with Dedicated Cross-linker Particles*

Nadine du Toit,[1,*] Kristian K. Müller-Nedebock,[1,2,†] and Giuseppe Pellicane[2,3,4,‡]

[1] *Department of Physics, Stellenbosch University, Stellenbosch 7602, South Africa*
[2] *National Institute for Theoretical and Computational Sciences, Stellenbosch 7602, South Africa*
[3] *School of Chemistry & Physics, University of KwaZulu-Natal, Pietermaritzburg, Scottsville 3209, South Africa*
[4] *Dipartimento di Scienze Biomediche, Odontoiatriche e delle Immagini Morfologiche e Funzionali, Università degli Studi di Messina, I-98125 Messina, Italy*

(Dated: February 2, 2026)

CONTENTS

I. Analytical model 1
A. Minimum repulsive potential 1
1. Strong intra-species cross-linking 1
2. Strong inter-species cross-linking 2

II. Molecular dynamics simulations 2
A. Reversible bonds 3
B. Dynamic structure factors using Dynasor 4

References 7

How to read this file. This Supplementary Information (SI) provides extended simulation details and additional figures. Interpretation of results is given in the main text. References and notation are consistent with the main paper unless otherwise stated.

I. ANALYTICAL MODEL

A. Minimum repulsive potential

The main text provides an expression to be solved for the minimum repulsive potential that needs to be incorporated in order to maintain stability of the system. The discussion below shows the values of this potential in each case for the chosen parameters in the plots of the main text.

1. Strong intra-species cross-linking

For the scenarios where there is only intra-species cross-linking, only cross-linkers and polymers of type A are considered, therefore the potentials involving polymers of type B vanish, *i.e.* $w_{\mathrm{B}} = v_{\mathrm{A,B}} = v_{\mathrm{B},\rho} = 0$. Consequently, the expression for the minimum repulsive potential may be simplified for intra-species cross-linking. Recalling eq. (**??**) this leads to

$$v_{\min} = \frac{1}{2}\left(\sqrt{4v_{\mathrm{A}.\rho}^2 + w_{\mathrm{A}}^2 - 2w_{\mathrm{A}}w_\rho + w_\rho^2} - w_{\mathrm{A}} - w_\rho\right) . \quad (1)$$

Note that each of the potentials w_{A}, w_ρ and $v_{\mathrm{A},\rho}$ is now a function of k and therefore so is $v_{\min}$. Consider the minimum repulsive potential to retain stability graphed in Fig. 1, as a function of k. A strong dip is observed around $k = 2$ after which the value plateaus at $v_{\min} = 1$. This suggests that at small and large length scales, a finite repulsive potential is required to retain stability of the system, whilst at intermediate length scales, where the dip is observed around $k = 2$,

* Corresponding author: 24461989@sun.ac.za
† kkmn@sun.ac.za
‡ gpellicane@unime.it

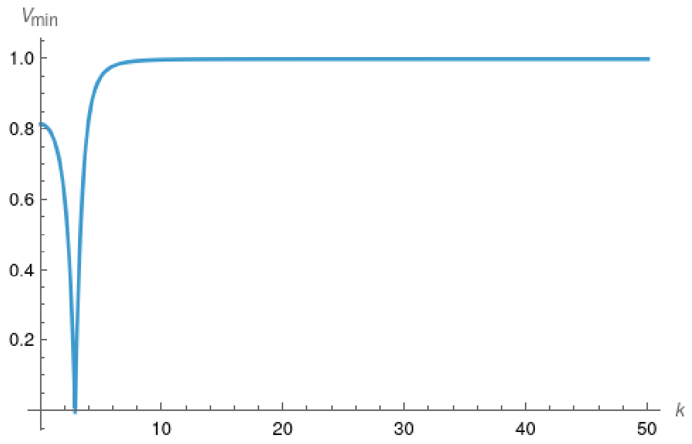

FIG. 1: Minimum repulsive potential as a function of k for strong intra-species cross-linking with $\epsilon = 0$, $\mu = 50$, $\bar{\rho}_0 = 0.25$, $\bar{C}_A = 1$, $\bar{C}_B = 1$, $\gamma = 1$, $\kappa = 1$, $\lambda = 1$, $\gamma_A = 1$, $\gamma_B = 1$, $\alpha = 1$, $\tau = 1$, $L_A = 100$ and $L_B = 100$.

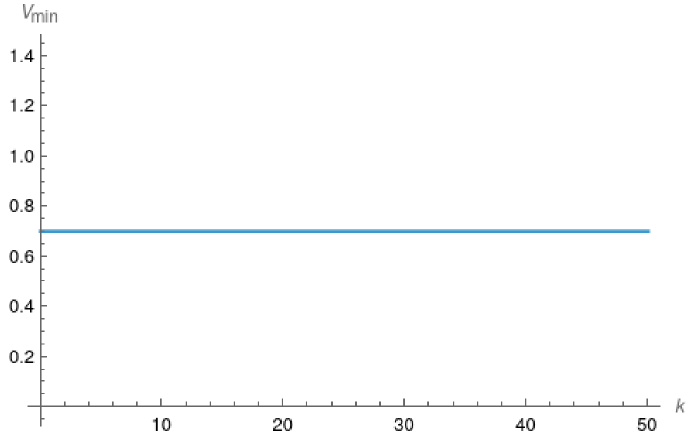

FIG. 2: Minimum repulsive potential as a function of k for strong inter-species cross-linking with $\epsilon = 50$, $\mu = 0$, $\bar{\rho}_0 = 0.5$, $\bar{C}_A = 1$, $\bar{C}_B = 1$, $\gamma = 1$, $\kappa = 1$, $\lambda = 1$, $\gamma_A = 1$, $\gamma_B = 1$, $\alpha = 1$, $\tau = 1$, $L_A = 100$ and $L_B = 100$.

the system retains stability without the repulsive potential. It should be noted here, that the k dependent value for $v_{\min}$ arises due to the approximation for the extension of the cross-linker particles. Due to the lengthy expressions for the potentials and $v_{\min}$, this was only investigated numerically here. It is likely that this length scale associated with the vanishing value of $v_{\min}$ is a numerical artifact due to the cross-linker extension approximation rather than a physically relevant phenomenon. In the implementation in the main text, this approximation is not be incorporated and so the $k = 0$ value is only relevant. Thus $v = 2$ has been chosen such that $v > v_{\min}$.

2. *Strong inter-species cross-linking*

For the strong intra-species cross-linking case the minimum repulsive potential required to maintain stability of the system is shown in Fig. 2. Since the higher order terms required to obtain the k-dependence have not included in this case, the minimum repulsive potential has a constant value of about 0.7 for the chosen parameters. Thu again selecting $v = 2$ satisfies $v > v_{\min}$.

II. MOLECULAR DYNAMICS SIMULATIONS

The main text includes molecular dynamics simulations of a two polymer system with reversible cross-linking. Whilst most details of the simulations are discussed in the main text, some additional details regarding the details

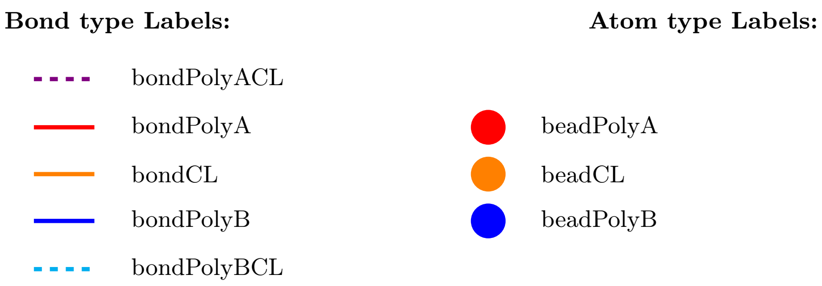

FIG. 3: Legend for molecule diagrams showing bond and atom type labels indicated by different coloured lines and filled circles.

of additional packages such as REACTER[1] and Dynasor [2] are given here. Details of the reversible bonding reactions implemented in the molecular dynamics simulations between polymer and cross-linker particles are discussed in Sec. II A. The analysis of the trajectories obtained during these molecular dynamics are discussed in Sec. II B.

A. Reversible bonds

Reversible bonding between polymer beads and cross-linkers were implemented using REACTOR [1] via the LAMMPS command '*fix bond/react*', in a two step process. This required specifying molecule templates both pre- and post-reaction, specifying the bonds and angles of the molecule such that bonds can be formed or broken accordingly. Along with the pre- and post-reaction molecule templates, a map file is required specifying the atom IDs of the initiator atoms, between which bonds will be formed or broken during the reaction, all atoms that are involved in the reaction and finally the atom IDs of edge atoms, which may be bonded to other atoms that are not part of the molecule template.

The pre-and post-reaction molecule templates and steps are illustrated in Figs. 4–6. In order to be able to discern between intra- and inter-chain cross-links, the monomers of each of the polymers were assigned different atom type labels '*beadPolyA*' and '*beadPolyB*' and bond type labels '*bondPolyA*' and '*bonddPolyB*' as shown in the colour coded legend in Fig. 3, although they were given the same properties and treated with the same parameters. Differentiating between atom types in this way, there are two ways in which bonds can form according to step 1, either between atom type '*beadPolyA*' and '*beadCL*' to form a bond of type '*bondPolyACL*' or between atom type '*beadPolyB*' and '*beadCL*' to form a bond of type '*bondPolyBCL*' as depicted in Fig. 4. Switching the pre- and post-reaction templates, the reverse of each of these reactions is also specified such that cross-linkers that are bound at one end can detach and diffuse freely throughout the system. As for the second step, there are four possible bond creation reactions and their four reverse reactions as depicted in Figs. 5–6. Figure 5 shows the formation and breaking of inter-chain cross-links. Here a cross-linker already has a bead that has a bond with a monomer on either polymer A or polymer B. The second bead of the cross-linker bonds, during step 2, with a monomer on the other polymer type. The reverse of this reaction allows one of the bonds between a monomer and cross-linker that forms an inter-chain cross-link, to break such that the cross-linker remains bound only at one end. Similarly, Fig. 6 depicts the two bond forming and two bond breaking reactions pertaining to intra-chain cross-links. In total there are thus 12 possible reactions that are implemented by the '*fix bond/react*' command in the simulation—one reaction corresponding to each arrow in Figs. 4–6—with 4 possible reactions in step 1 and its reverse and 8 reactions in step 2 and its reverse. At each time step each of these reactions is attempted with a specified probability. For a reaction to be attempted, initiator atoms (given by atom IDs 3,6,7 and 10 in Figs. 4–6) are identified according to the pre- and post-reaction templates and these atoms must be within a specified distance from one another. The reaction distances and probabilities for each step of the reaction are given in Table I. After each successful reaction, *i.e.* bond formation or breakage, a stabilisation period of 100

Reaction	Minimum distance	Maximum distance	Reaction probability
Step 1	0.0	1.6	0.8
Step 1 reverse	1.1	2.5	0.05
Step 2	0.0	1.6	0.95
Step 2 reverse	1.1	2.5	0.05

TABLE I: Reaction distances and probabilities for the steps of *'fix bond/react'*.

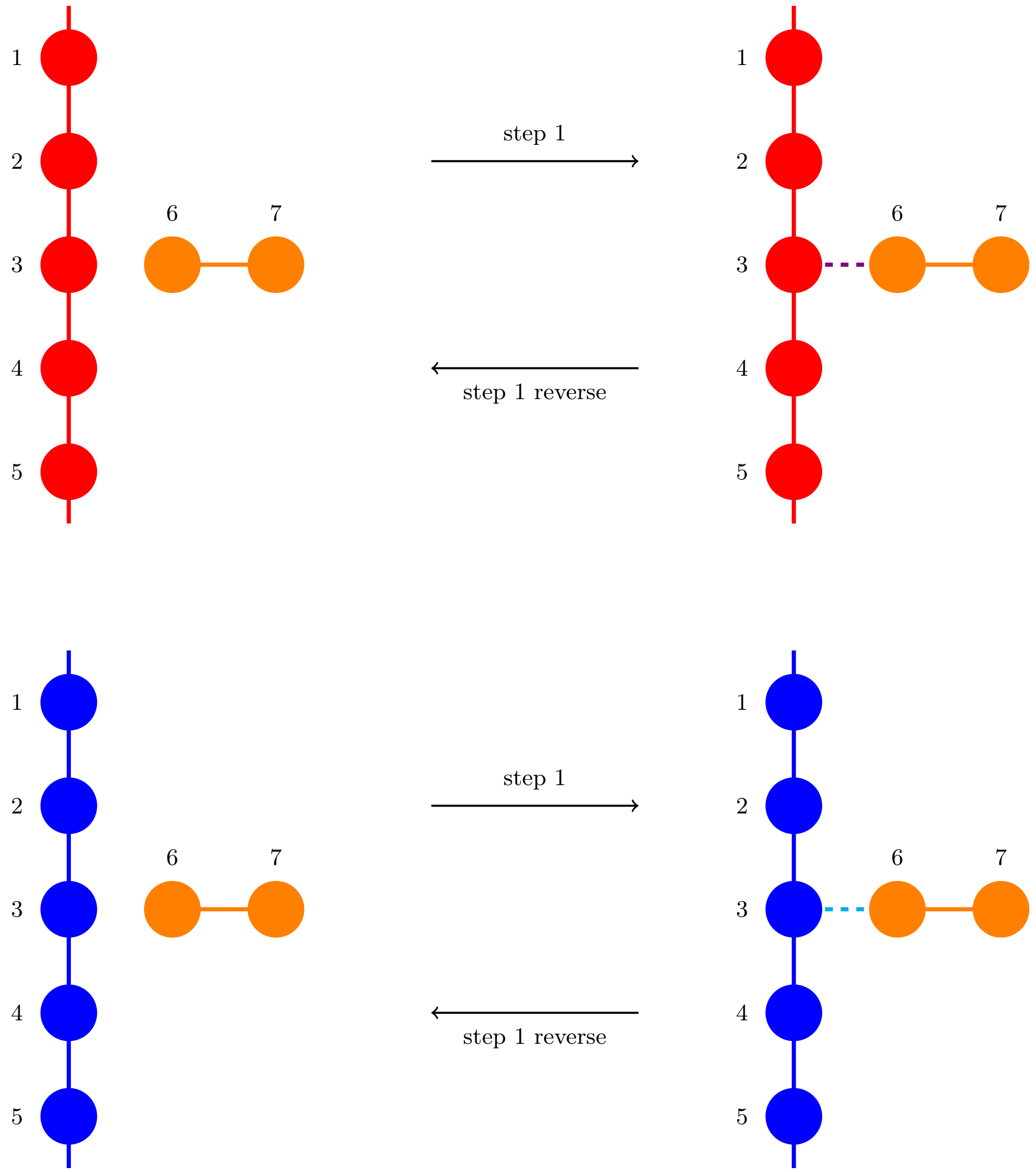

FIG. 4: Molecule reaction templates and four possible reactions (indicated by arrows) for step 1 of '*fix bond/react*'. Atom IDs 1-7 are indicated next to each atom. Bonds and atoms are colour coded according to the legend in Fig. 3. For step 1, Atom IDs 1 and 5 are edgeIDs.

time steps is applied to avoid immediate rebonding or rebreaking. During this time, atoms involved in the reaction are integrated via an internal '*fix nve/limit*', with a maximum displacement of 1.0 (in reduced units). This relatively large value allows atom motion to proceed without artificial restriction, with stabilisation serving primarily to prevent repeated reactions on short timescales rather than suppressing post-reaction forces.

B. Dynamic structure factors using Dynasor

After loading the LAMMPS trajectory files into Dynasor, spherically distributed q-points were generated corresponding to the trajectory with a maximum q-value of 6 and the maximum number of q-points set to 170 000. The q-points were filtered to remove values smaller than $q = \frac{2\pi}{L}$, where $L = 100$ is the length of the box, so as to avoid evaluations of the dynamic structure factor on length scales irrelevant to the simulation. This resulted in the sample of q-points shown in the histogram in Fig. 7, where the sample has been divided into 50 bins.

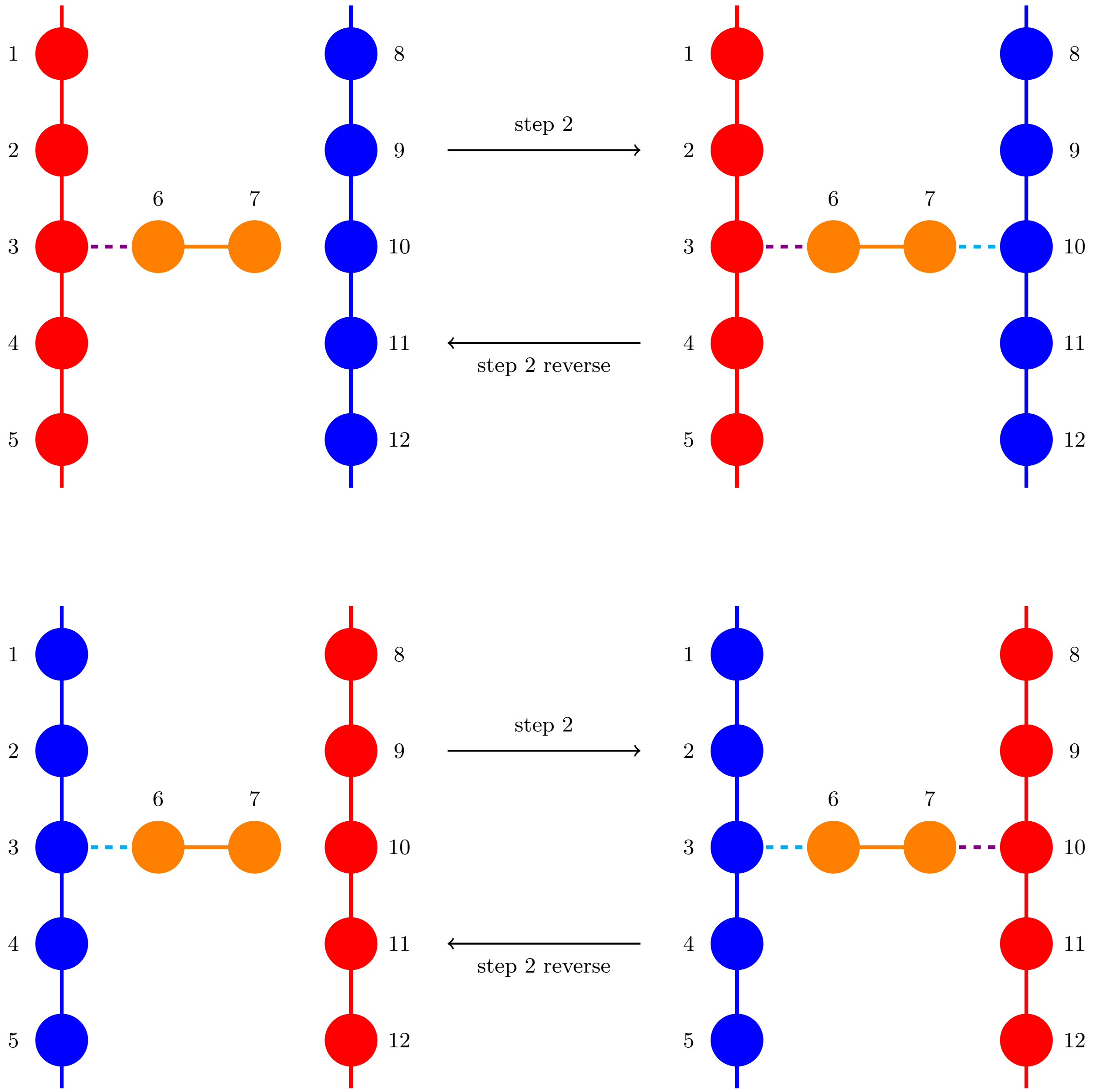

FIG. 5: Molecule reaction templates and four possible inter-chain reactions (indicated by arrows) for step 2 of *fix bond/react*. Atom IDs 1 -12 are indicated next to each atom. Bonds and atoms are colour coded according to the legend in Fig. 3. For step 2, Atom IDs 1, 5 8 and 12 are edgeIDs, whilst atom IDs 3 and 6 are the initiator atom IDs.

The q-points in Fig. 7 and the previously loaded trajectory, were passed to the Dynasor function to compute the dynamic structure factors, with a window size of 100 and a timestep of 0.05, corresponding to the output interval of the LAMMPS simulation. This resulted in a Dynasor sample containing the dynamic structure factors evaluated for the array of q-points and an associated frequency axis consisting of 101 ω-values. As a final post-processing step,via a Dynasor function the results were spherically averaged over q-points with 50 bins to remove some of the noise. The dynamic structure factors obtained in this manner for both simulations with and without cross-linking are shown in Fig. 8. These figures are the 3D plots corresponding to the 2D slices in the main text.

Figures 8a and 8b, show the dynamic structure factors for polymers of type A with and without cross-linking, respectively, whilst Figs. 8c and 8d show the same for polymers of type B. All four plots show the usual shape of a peak at small q and ω-values that goes to zero at larger q and ω. The plots of the dynamic structure factors for the polymers with cross-linking, retain a similar shape but have noticeably broader peaks. The broadened peaks are especially prominent at $\omega = 0$, indicating that fluctuations in polymer densities remain correlated on smaller length scales in the long time limit. This also corresponds to the overall qualitative trend of cross-linking resulting in

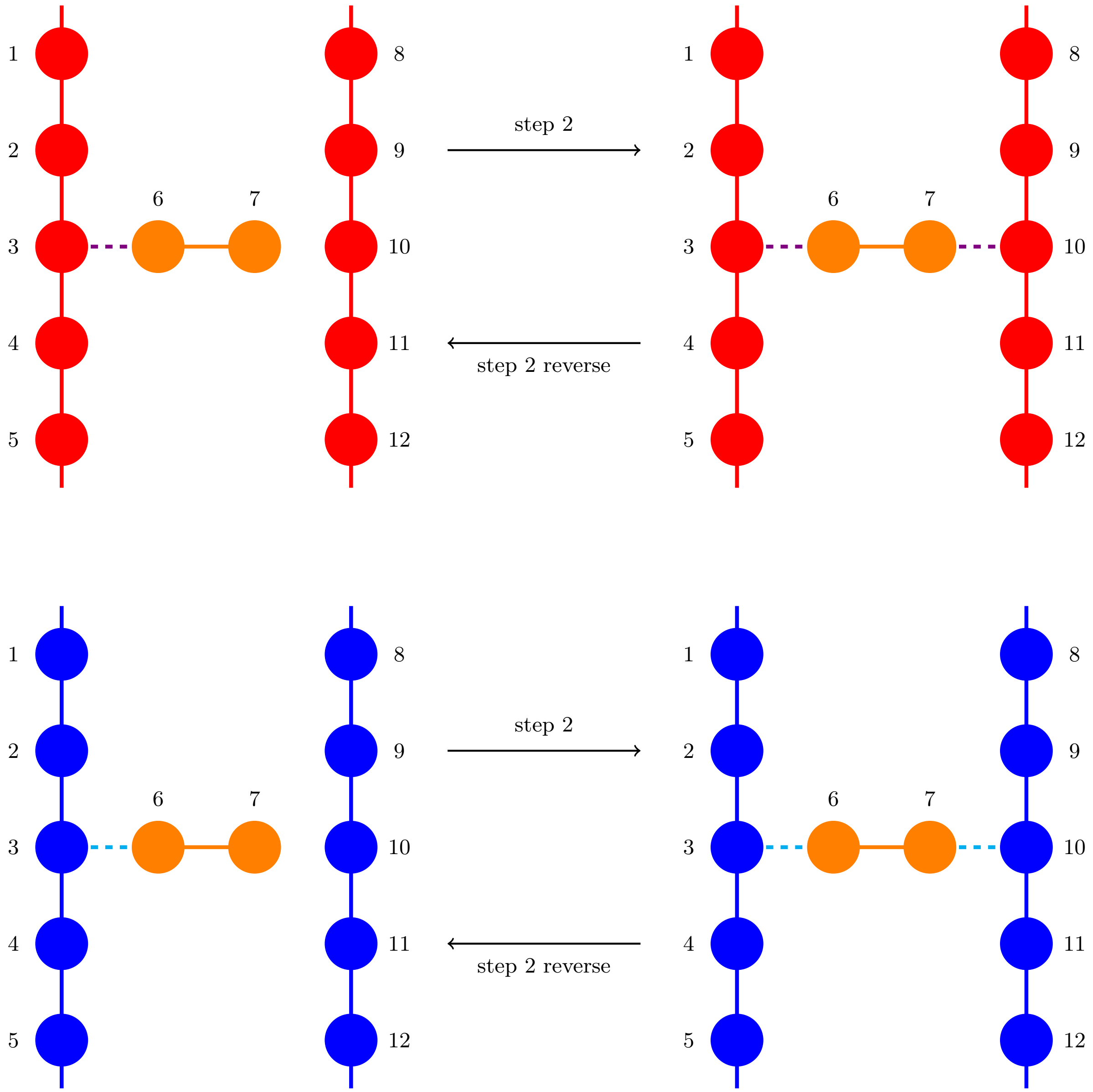

FIG. 6: Molecule reaction templates and four possible intra-chain reactions (indicated by arrows) for step 2 of '*fix bond/react*'. Atom IDS 1 -12 are indicated next to each atom. Bonds and atoms are colour coded according to the legend in Fig. 3. For step 2, Atom IDs 1, 5 8 and 12 are edgeIDs.

broadening of the peaks of the dynamic structure factors, observed in the results of the analytical work presented in the main text.

Figures 8e and 8f show the dynamic structure factors for the cross-linker, with and without cross-linking , respectively. Again, the peak at small q and ω is observed, with a noticeable broadening in the peak, especially along the q axis, with cross-linking. The peak of the dynamic structure factor also extends to significantly higher values at small q and ω in the cross-linking case in Fig. 8e, indicating that a significant increase in the correlation of density fluctuations of the cross-linkers at large length and time scales. The observed feature makes sense, since majority of the cross-linkers are bound to polymers at any given time throughout the simulation, and this should result in an increase in collective motion of the cross-linkers.

Finally, the dynamic structure factors can be investigated and compared for cross-correlations between polymers of different types and cross-linkers. This is shown in Fig. 9. The cross-correlations for the simulations without cross-linking are shown in Figs. 9b, 9d and 9f. These all show strong anti-correlations at large length and time scales that decay to zero at smaller length and time scales. The anti-peaks are narrower in q for the cross-correlations

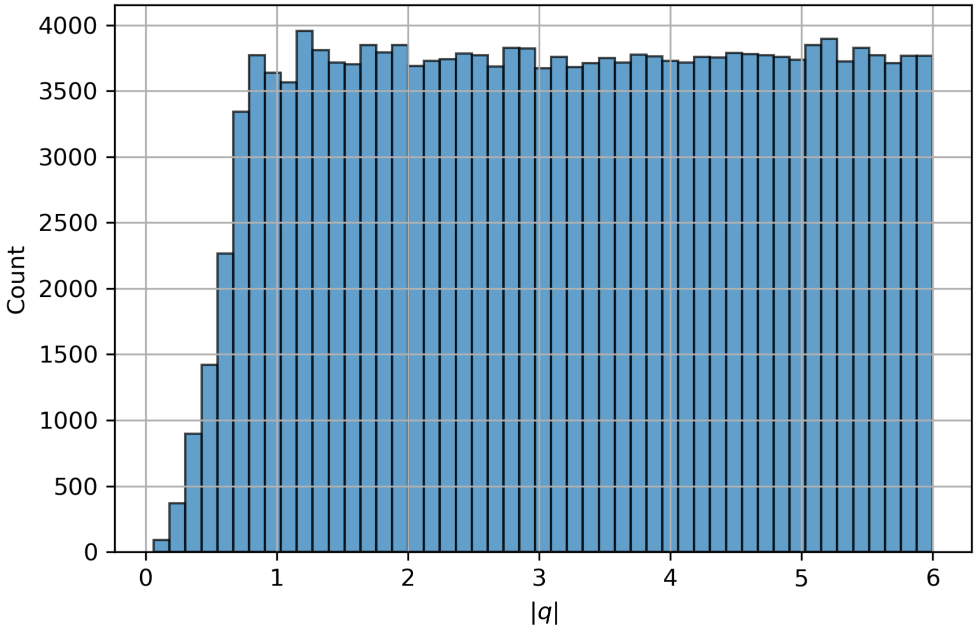

FIG. 7: Distribution of the 170 011 q-points used for calculating dynamic structure factors

between polymers and cross-linkers in Figs.9d and 9f than between the two different types of polymers in Fig. 9b, indicating a stronger anti-correlation between polymers than between polymers and cross-linkers. This corresponds to what is expected in the simulations with no linking, since the only interaction between particles are the repulsive Lennard-Jones potentials discussed in the main text.

For the simulations with cross-linking, the cross-correlations are shown in Figs. 9a, 9c and 9e. These all show strong positive peaks at small q and ω, with varying magnitudes of anti-peaks indicating negative correlations at slightly larger q-values where $\omega = 0$. For the cross-correlation between different polymer types in Fig. 9a, the anti-peak is the largest and occurs between $q = 0$ and $q = 1.0$. This indicates that, on long time scales, density fluctuations of polymers of different types are anti-correlated with one another on intermediate length scales, but become strongly correlated with one another on very large length scales near $q = 0$, with no correlation on very short length scales. A similar pattern is observed in the cross-correlations between the cross-linkers and polymers in Figs. 9c and 9e, but with a broader peak corresponding to strong correlations at long length scales followed by a very slight negative dip indicating anti-correlations around $q = 2$, with no correlations at smaller length scales. Thus density fluctuations of polymers and cross-linkers, at long time scales, are strongly correlated at large length scales and slightly anti-correlated on intermediate length scales with no correlations at short length scales.

[1] Jacob R. Gissinger, Benjamin D. Jensen, and Kristopher E. Wise. REACTER: A Heuristic Method for Reactive Molecular Dynamics. *Macromolecules*, 53(22):9953–9961, November 2020.

[2] Erik Fransson, Mattias Slabanja, Paul Erhart, and Göran Wahnström. Dynasor—A Tool for Extracting Dynamical Structure Factors and Current Correlation Functions from Molecular Dynamics Simulations. *Advanced Theory and Simulations*, 4(2):2000240, 2021.

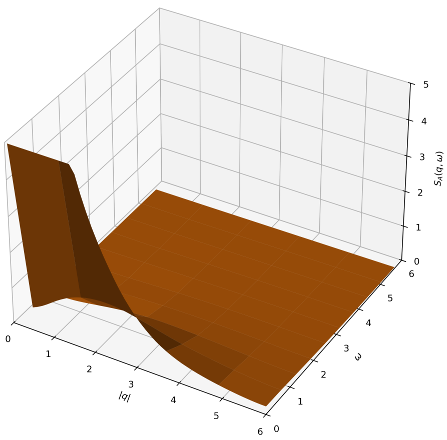

(a) $S_{\rm A}(q,\omega)$ with cross-linking

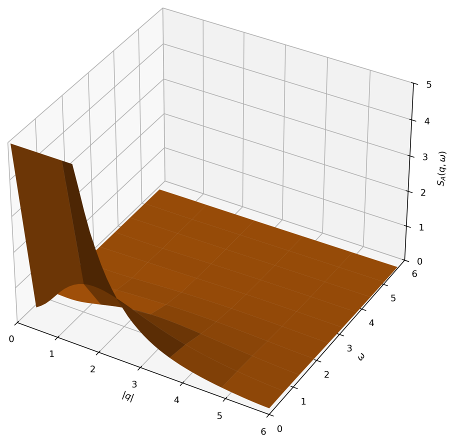

(b) $S_{\rm A}(q,\omega)$ without cross-linking

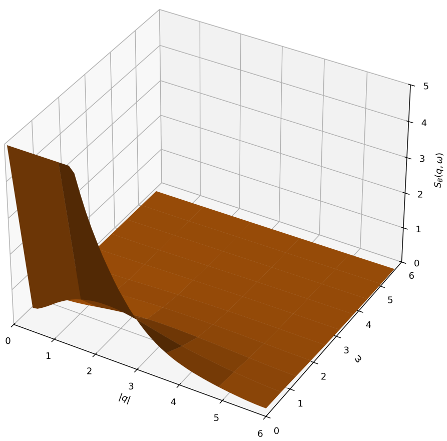

(c) $S_{\rm B}(q,\omega)$ with cross-linking

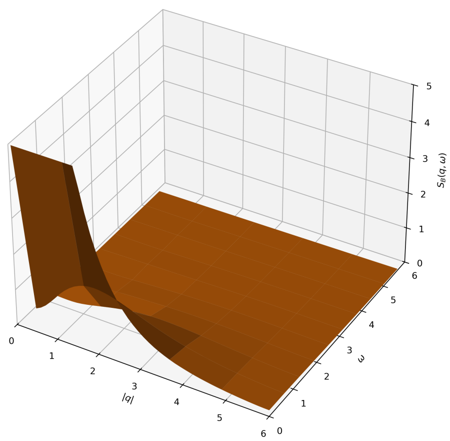

(d) $S_{\rm B}(q,\omega)$ without cross-linking

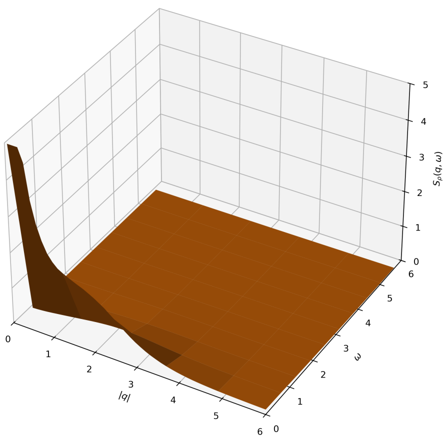

(e) $S_\rho(q,\omega)$ with cross-linking

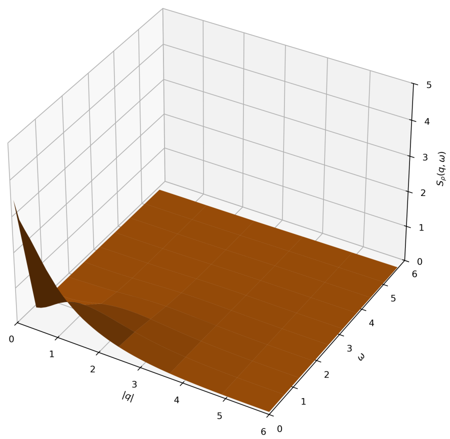

(f) $S_\rho(q,\omega)$ without cross-linking

FIG. 8: Dynamic structure factors from polymer network simulations with and without cross-linking.

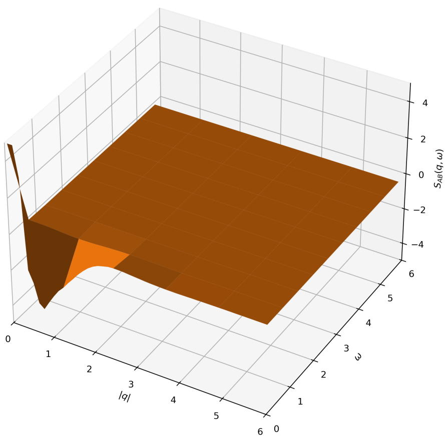

(a) $S_{\mathrm{AB}}(q,\omega)$ with cross-linking

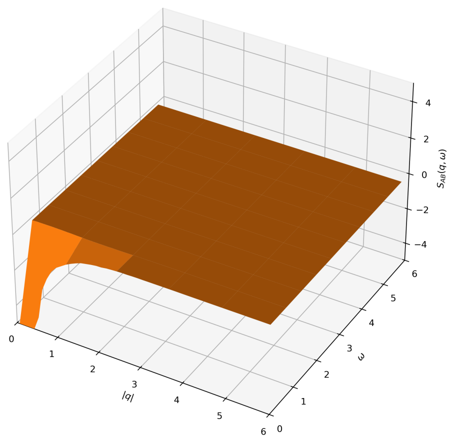

(b) $S_{\mathrm{AB}}(q,\omega)$ without cross-linking

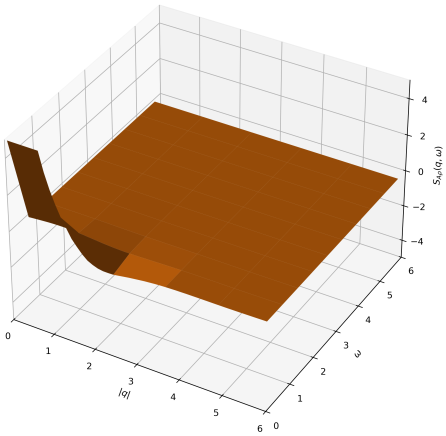

(c) $S_{\mathrm{A}\rho}(q,\omega)$ with cross-linking

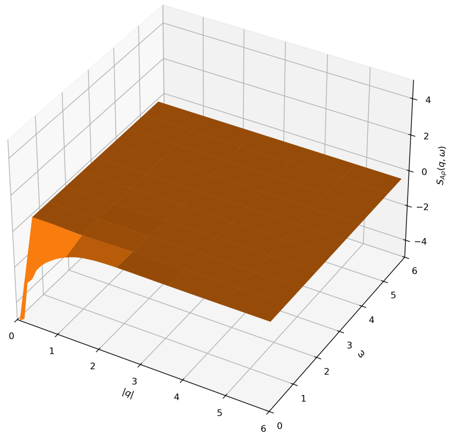

(d) $S_{\mathrm{A}\rho}(q,\omega)$ without cross-linking

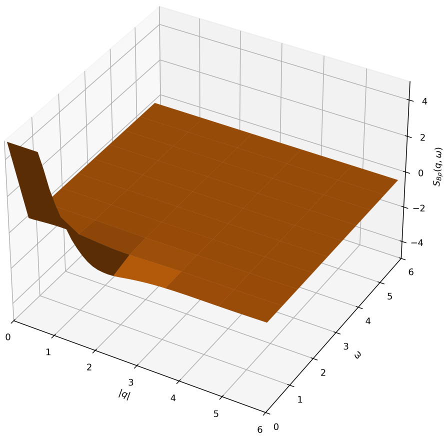

(e) $S_{\mathrm{B}\rho}(q,\omega)$ with cross-linking

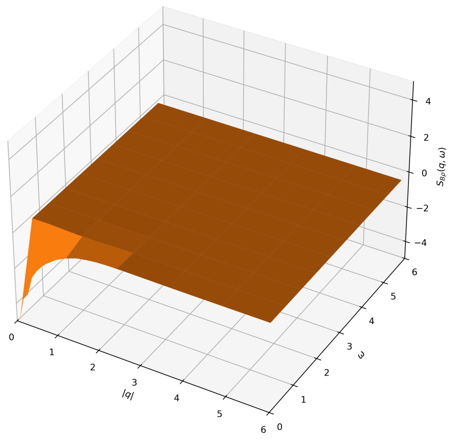

(f) $S_{\mathrm{B}\rho}(q,\omega)$ without cross-linking

FIG. 9: Dynamic structure factors for the cross-correlations from runs with and without cross-linking.
